# Analytical Methods for the Detection and Quantification of ADCs in Biological Matrices

**DOI:** 10.3390/ph13120462

**Published:** 2020-12-14

**Authors:** Héloïse Cahuzac, Laurent Devel

**Affiliations:** Département Médicaments et Technologies pour la Santé (MTS), CEA, INRAE, SIMoS, Université Paris-Saclay, 91191 Gif-sur-Yvette, France; heloise.cahuzac@cea.fr

**Keywords:** antibody–drug conjugates, pharmacokinetics and biodistribution, liquid chromatography coupled to mass spectrometry, ligand-binding assays, in vivo imaging, ex vivo autoradiography

## Abstract

Understanding pharmacokinetics and biodistribution of antibody–drug conjugates (ADCs) is a one of the critical steps enabling their successful development and optimization. Their complex structure combining large and small molecule characteristics brought out multiple bioanalytical methods to decipher the behavior and fate of both components in vivo. In this respect, these methods must provide insights into different key elements including half-life and blood stability of the construct, premature release of the drug, whole-body biodistribution, and amount of the drug accumulated within the targeted pathological tissues, all of them being directly related to efficacy and safety of the ADC. In this review, we will focus on the main strategies enabling to quantify and characterize ADCs in biological matrices and discuss their associated technical challenges and current limitations.

## 1. Introduction

Antibody–drug conjugates (ADCs) consist of a small-molecule drug (payload) covalently bound to a monoclonal antibody via a chemical linker. By merging the specific binding properties of antibodies to the potency of small molecules, ADCs are designed to selectively deliver cell-killing agents to targeted pathogenic tissues, while limiting systemic toxicity. Most of the ADCs developed so far are potential anticancer agents and nine of them were approved by the Food and Drug Administration (FDA) for the treatment of various types of cancer (Table 1). Many other ADCs are currently in advanced clinical trials not only for anticancer applications [1,2,3,4,5] but also to treat intracellular *Staphylococcus aureus* bacterial infection [6], for the targeted delivery of kinase inhibitors [7], as well as ADCs with anti-inflammatory properties [8,9].

In most approved and clinical-stage ADCs, the toxic payloads are microtubule disruptors or DNA-damaging agents [10,11]. In the first and second generations of ADCs, those payloads were conjugated to antibody component in a stochastic manner, leading to heterogeneous mixtures of chemically distinct molecules varying in both drug-to-antibody ratio (DAR) and conjugation sites [1]. This last decade, with the aim to better control the position and the number of drug loads, new conjugation strategies were developed to access a new generation of ADCs with improved homogeneity [1,2,12,13]. Those site-specific reactions rely on the insertion of engineered cysteine residues [14] or unnatural amino acids within the antibodies core [15,16], enzymatic conjugation [17,18,19], cross-link of the reduced interchain disulfides with rebridging chemical reagents [20], and glycan-mediated conjugation [21]. The conjugating linkers between payload and antibody are generally classified into cleavable and noncleavable ones, which can behave quite differently in a biological system [22]. There are several linker types comprising hydrazone [23,24], disulfide [25], dipeptide [26,27], tetrapeptide [28], glucuronide [29], phosphate-ester [30], or noncleavable linker [31], which are sensitive to different triggers such as pH variation, modification of the reducing environment, or local presence of enzymes. Noteworthy, enzymatically cleavable peptide-base linkers have been designed both to be highly stable in circulation and to exhibit a more specific drug release mechanism within the targeted tissues [32]. It was also demonstrated that the in vivo stability of the linker during circulation depends considerably on the location and solvent accessibility to the conjugation site [14].

**Figure 1:**
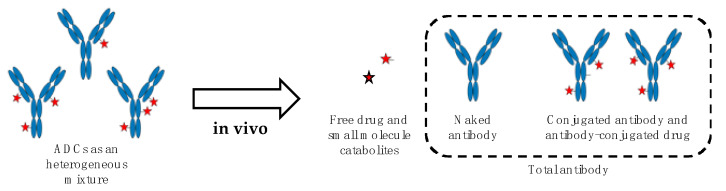
The catabolic fate of ADCs increases the complexity of the mixture in vivo giving rise to multiple analytes to be measured. The antibody is colored in blue, the free drug and its metabolites are represented as red stars.

Like for other therapeutics, the understanding of ADCs fate in vivo is critical for their development, optimization, and successful transfer to human. The pharmacology of ADCs is complex and several factors potentially impacting their pharmacokinetics, toxicity, and biodistribution have to be carefully considered. This notably includes their physical characteristics (e.g., composition and size, surface engineering, DAR, conjugation site, and net charge at physiological pH) and their propensity to generate antidrug antibodies (ADAs), the latter correlating with ADCs immunogenicity. All those factors have been recently reviewed [33,34] and are not covered by the present review. In any event and due to their multicomponent nature, ADCs must be considered as a complex and dynamically changing mixture in vivo, driven by undesirable drug release [35], catabolism [36], and variable clearance rate according to DAR [37]. Therefore, in such a scenario, multiple ADCs-derived analytes have to be measured to properly describe the stability and biodistribution of ADCs as well as the amount of drug accumulated within the targeted tissues. The main ADCs-derived analytes comprise the naked antibody, the conjugated antibody, and antibody-conjugated drug, all the three corresponding to total antibody, and the released small molecule drug as well as its metabolites [36,38,39,40] (Figure 1).

To determine systemic (e.g., in plasma) and local (e.g., in tissues) levels of ADCs-derived analytes, two main types of bioanalytical methods can be distinguished. The analytical methods that do not imply any prelabeling of the ADCs components and those requiring to label the antibody part, the payload or both of them. The label-free methods mainly rely on ligand-binding assays (LBAs), liquid chromatography coupled to mass spectrometry (LC–MS/MS), or a combination of both methods. Most of the time in the case of labeled ADCs, a fluorescent reporter or a radioactive isotope are incorporated within the structure of the antibody or/and the drug. In this case, the pharmacokinetics, biodistribution and tissues uptake of ADCs are determined either by noninvasive imaging approaches (e.g., positron emission tomography (PET) or fluorescence molecular tomography (FMT)) or by liquid scintillation counting and ex vivo radio-imaging after animal sacrifice, the latter being obviously restricted to preclinical studies. The present review proposes to outline those different bioanalytical methods and discuss their associated technical challenges aiming at characterizing and accurately quantifying the different ADCs-derived analytes in various biological matrices, with a particular focus on preclinical studies.

## 2. Label-Free Bioanalytical Methods

### 2.1. Ligand-Binding Assays (LBAs)

Out of all the LBA formats, ELISA (enzyme-linked immunosorbent assay) relying on an immunoaffinity detection of the protein of interest, is the gold standard of LBA for the analysis of large molecules in complex biological samples. This bioanalytical method offers unique advantages for the quantification of proteins, such as a high sensitivity and a broad range of quantification in a small biological volume without any sample extraction steps. However, the complex multicomponent structure of ADCs and their heterogeneous and dynamically changing nature in vivo present several analytical challenges for LBAs [36,41]. In this respect, the quantification of total antibody requires the use of detection reagents that bind selectively and efficiently to the antibody component. This includes generic anti-human antibody, protein A, G or L, or more specific detection reagent such as anti-idiotype antibody. This approach has been successfully applied to the quantification of several ADCs in various biological matrices, including anti-CD33-Calicheamicin ADC [42], T-DM1 [43], and mAb-MMAE conjugates [14,44,45,46]. LBAs can also utilize antidrug- specific antibody as a capture antibody to quantify conjugated antibody [47,48,49,50] or antidrug antibody as detection reagents [51,52,53,54]. Regarding the choice of primary antibody as capture agent and to avoid any potential interferences from endogenous IgGs, it is essential to evaluate its nonspecific cross-reactivity. For instance, it is recommended to choose antibodies with minimum reactivity to mouse and rat for preclinical studies. Both monoclonal and polyclonal antibodies are suitable as capture and detection agents. Monoclonal antibodies provide mono specificity toward a single epitope, which allows fine detection and quantitation of small differences in antigen. Polyclonal antibodies are used to capture as much antigen as possible, while monoclonal ones are used to detect ADCs with improved specificity. Polyclonal antibodies are much less expensive to produce than monoclonal antibodies. Therefore, an appropriate balance between cost and required specificity has to be systematically considered.

Importantly, conjugated antibody ELISA is very sensitive to the choice of the antidrug antibody, and therefore the key aspect in such assay development is to identify an antidrug antibody that is minimally influenced by the drug load. Indeed, it has been reported that the small molecule drug can indirectly influence the reagents binding to the antibody, resulting in species varying in DAR that are not detected with the same efficiency and accuracy. For instance, purified DAR fractions with high MMAE drug load were under-recovered (around 70% and 55% of recovery for DAR 6 and DAR 8, respectively) in a total antibody ELISA assay using various reagents [55]. As a consequence, the development of such an assay requires the screening of multiple formats and reagents in relevant biological matrices including plasma or serum [47]. Compared to conventional ELISA where reagents are added sequentially, the semihomogeneous assay (SHA), relying on premixing of capture and detection reagents with the sample solution, turns out more effective to accurately detect the whole set of ADCs regardless of their drug loading [55]. Finally, LBAs can also be suitable for the quantification of the drug moiety. Thus, both an anti-MMAE antibody and a HRP-MMAE were used to quantitate free MMAE released from ADC after an in vitro incubation with Cathepsin B [54].

However, LBAs with excellent specificity and accuracy remain difficult to achieve and require the use of efficient and selective capture antibodies. The development of such agents may turn out difficult and time-consuming, making those immunoassays sometimes unpractical in the early phase of ADCs development. Furthermore, their quantitative accuracy, specificity, and reproducibility can be compromised by interferences from endogenous IgGs and degradation of the antibody component. ELISA is very commonly used in quantification of ADCs in plasma but is often matrix- and species-dependent, rendering its transfer across different biological matrices (e.g., from plasma to tissues) sometimes challenging [36,41]. Nevertheless, these limitations can be overcome when the capture and the detection antibodies are finely selected. Thus, by using a total antibody anti-human IgG antibody (Fc-specific), trastuzumab-vc-MMAE can be sensitively and accurately dosed in both plasma and tissue homogenates, with a detection limit down to 1 ng/mL [56].

### 2.2. Hybrid Ligand-Binding Immuno-Affinity Capture Followed by Liquid Chromatography Coupled to Mass Spectrometry (LB-LC–MS)

The ligand-binding and LC–MS (LB-LC–MS) hybrid method that combines the strength of both technologies is becoming a gold standard approach in ADC bioanalysis [57,58,59]. In this respect, the ligand-binding affinity enrichment step allows to spare interfering abundant proteins while the LC–MS based method offers quantitative bioanalysis with multiple analytes detection with high specificity within a single sample. In preclinical samples, an immobilized anti-human Fc antibody is commonly used to isolate ADCs from animal plasma whereas a specific anti-idiotype antibody is required in human samples [60]. After washing steps and proteolytic digestion, the target antibodies are unambiguously identified through their MS/MS fragmentation pattern [61].

**Figure 2:**
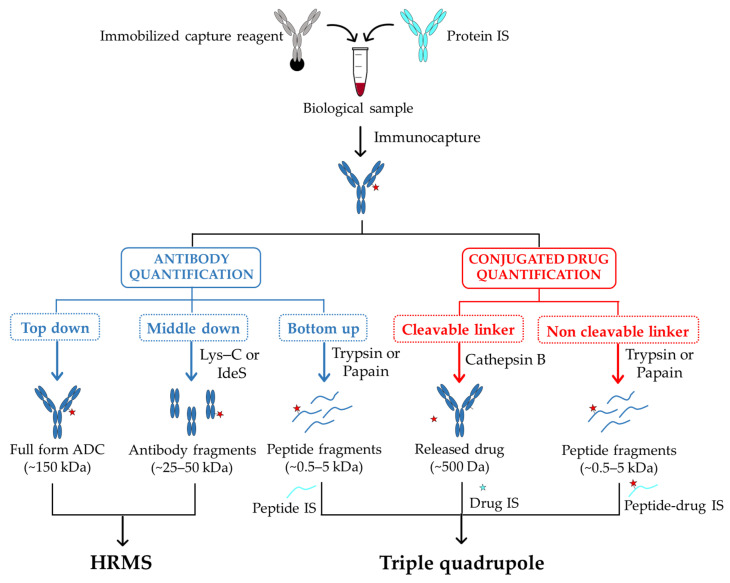
A typical workflow for the bioanalysis of ADCs in biological sample using LB-LC–MS/MS methods. The antibody and the peptide fragments are colored in blue, the free drug and its metabolites are represented as red stars. The internal standards, Peptide IS and Peptide-drug IS, are colored in cyan. The Drug IS is represented as a cyan star. IS: internal standard

The quantitative analysis is mostly carried out using the “bottom-up” strategy. In this case, a digestion of the captured ADC is followed by a LC–MS/MS analysis to detect and identify their selected signature peptides (Figure 2). The choice of the signature peptides within the antibody core mainly relies on the biological matrix in which ADCs have to be detected and quantified. In this respect, peptides from the constant region of antibody part will be considered as signature peptides for ADCs characterization in animal models. In human samples and to limit interferences with endogenous human IgGs, signature peptides from antibody variable regions will be preferred. Regarding the quantification, it is performed by comparing signature peptides of target antibody to standard peptides present at defined amounts. Such internal standards must be identical in chemical and physical properties to the peptides of interest, with small differences in mass that enables their discrimination by mass spectrometry. To this purpose, peptides labeled with stable isotopes ^13^C and ^15^N are commonly used [62]. In order to compensate potential variations during sample preparation, a stable-isotope-labeled antibody as internal standard can be also incorporated from the beginning of the enrichment process [63]. Although this workflow offers a very good sensitivity of quantification, the signature peptides only provide limited information about potential structural changes of the entire ADC and may therefore lead to a misestimation of their concentration in sample solutions.

To overcome these limitations, the “top-down” strategy that allows assessing qualitative and quantitative changes simultaneously, has come out as a complementary analysis to the “bottom-up” approach. In this case, the whole target protein is analyzed using high-resolution mass spectrometry (HRMS) with no further digestion step required [64] (Figure 2). This approach has been used to efficiently quantify intact antibodies in biological samples [65,66], and variations resulting from proteolytic digestion step remain limited. Nevertheless, compared to peptide level quantification, LC–MS quantification of intact ADCs presents many other challenges. Indeed, due to their larger size and heterogeneity, their mass signal can be dispersed among multiple charge states, resulting in a loss of detection sensitivity [67]. Further, antibody internal standards are almost systematically required for the absolute quantification of ADCs [65]. In some cases, this turns out problematic when the internal standard coelutes with the target specie, thus contributing in complicating the spectra deconvolution [67]. However, by applying a narrow-window of extracted-ion chromatogram (EIC) and a deconvolution method with no internal standard, an accurate quantification with an acceptable detection threshold (5 µg/mL) can be achieved [67]. When a high-resolution accurate-mass (HR/AM) mass spectrometry is used, this “top-down” strategy enables to characterize the in vivo biotransformations of trastuzumab emtansine (T-DM1 or Kadcyla) in circulation in tumor-bearing mice [68]. In this case, by combining an affinity capture with a biotinylated HER2 extracellular domain (EDC) and mass analyses on total antibody using a HR/AM orbitrap, the authors provided new data on T-DM1 catabolism, including maleimide exchange, loss of payload, and linker-drug hydrolysis.

A good compromise between high sensitivity and the access to dynamic changes of ADCs in biological matrixes can be obtained with a “middle-down” approach implying ADCs digestion into smaller subunits (subfragments) before LC–MS analysis (Figure 2). In this case, a partial digestion with Lys-C of a protein A bound IgG allows the direct characterization of the released Fab fragment (50 kDa), and its accurate quantification [69]. An additional step of disulfide bridges reduction after digestion using the endopeptidase IdeS has also been reported to generate three approximately 25 kDa fragments (Lc, Fc/2, Fd) that can be directly analyzed by LC–MS [70]. In all cases, the reduced size of analytes results in an improved sensitivity and resolution of mass spectra compared to the intact ADC.

In a comparable manner to LBAs, one of the main limitations of affinity capture combined with LC−MS is that it requires specific capture reagents capable of selectively bind the targeted ADCs, as well as its derived analytes. In addition, the majority of LB-LC–MS focus on Fab-conjugated ADCs and are not readily applicable to Fc-conjugated ones. In such a context, a generic and universal affinity capture LC−MS assay has been recently developed to evaluate biotransformation of ADCs in preclinical studies, independently of the antibody type, conjugation site (Fab, Fc), or conjugation technologies [71]. In this case, the ADCs are captured from mouse serum with generic antihuman F(ab′)2 coated beads and, depending on the site of conjugation, either directly eluted or digested “on-bead” with IdeS enzyme before elution. The resulting ADC sub fragments are finally analyzed by LC−HRMS. The advantages of this method are that it can be performed using commercially available generic reagents and requires sample preparation time of less than 7 h.

The hybrid immuno-affinity capture LC–MS is also suitable for the quantification of the conjugated payload [72]. The characteristic of the linker moiety mainly determines the sample processing strategy for the conjugated payload analysis. In the case of ADC incorporating an enzyme- sensitive dipeptide Val-Cit linker (e.g., brentuximab vedotin), the release of payload is performed by digestion with proteases such as cathepsin B or papain [54,72,73,74,75]. For noncleavable linkers, the whole antibody is first digested by a protease and the resulting drug-conjugated peptide is the surrogate analyte to monitor by LC–MS [76].

Interestingly, this LB-LC–MS/MS method has also been reported for the quantitative assessment of albumin linker-payload adduct. This mainly encompasses thiol-maleimide based ADCs that can undergo deconjugation through a thiol exchange with the Cys34 from the serum albumin in circulation [52]. In this case, the capture reagents are antipayload [77] or an antialbumin antibodies [78].

### 2.3. Nano-Surface and Molecular-Orientation Limited (nSMOL) Proteolysis

Shotgun proteomics enables quantification of fully digested peptides by trypsin using liquid chromatography MS (LC/MS). With the aim to apply a comparable technology to monoclonal antibodies and ADCs quantitation in biological matrices, Shimada and coworkers recently developed an elegant approach consisting in limiting protease access to a specific region of antibody substrate, thus enabling the generation of a restricted array of peptides fragments (signature peptides) subsequently analyzed by mass spectrometry [79,80]. This method, named nano-surface and molecular orientation limited (nSMOL) proteolysis, allows the selective and orientational proteolysis of the antibodies Fab region and relies on three distinct critical steps: (i) the miniaturization of the reaction field while increasing the surface area of the protease reaction through the use of nanoparticles, (ii) the antibody immobilization onto resin via protein A/G affinity binding, and (iii) the Fab region-selective proteolysis by limiting protease access to the substrate by tuning the particle size (protease) and the pore diameter of the resin immobilizing the antibodies. This approach has notably permitted to quantify Trastuzumab emtansine (T-DM1) in human plasma [80]. In the same study, by using nSMOL for the antibody and polarity-selective liquid–liquid partition for the cytotoxic drugs, it was possible to obtain an overall picture of antibody–drug conjugate T-DM1 pharmacokinetics and released payloads in samples from patients with breast cancer. Noteworthy, nSMOL proteolysis also enables to accurately quantify antibody drug in coexistence with antidrug antibodies (ADAs) [81].

### 2.4. MS Methods to Assess Pharmacokinetics and Biodistribution of Payload

Conventional small-molecule LC–MS/MS assay is designed to measure drug that is no longer bound to the antibody. This includes the portion released during circulation phase as well as that liberated during ADC catabolism. A step of protein precipitation or solid phase extraction (SPE) is systematically performed to remove all the plasma proteins prior to LC analysis. Molecular and fragments masses are used to determine the structure of the major metabolites and UV absorption at characteristic wavelength enables the quantification of different entities. The sensitivity of the assay is one of the major challenges associated with the free drug quantification since the unconjugated drug can be present at a very low concentration in biological samples. For instance, it has been reported that the quantification of the free DM1 payload in clinical samples of patients treated every 3 weeks was not possible as the signal was below the lower limit of quantification (LLOQ) of the method (0.20 ng/mL) [82]. In another study, the quantification of the unconjugated DM4 has only been performed during the first 24 h of a 3-week study using a LC–MS/MS method with a LLOQ of 0.50 ng/mL [83]. However, the method sensitivity remains difficult to predict since it depends both on the payload considered and the performances of mass spectrometry device used.

Matrix assisted laser desorption/ionization imaging mass spectrometry (MALDI–IMS) can be also used to directly detect the distribution of payload within tumor tissues [84]. In this study, the authors conducted quantitative and semiquantitative analyses of MMAE in subcutaneous tumors by using LC–MS/MS and MALDI–IMS, respectively. Interestingly, by exploiting MALDI–IMS conditions dedicated to low-molecular weight substances, they visualized only free MMAE without interference from conjugated MMAE. Further, by relying on a specific MS pattern of the MMAE payload, the latter can be observed with high reliability and accuracy in tumor tissues.

**Figure 3:**
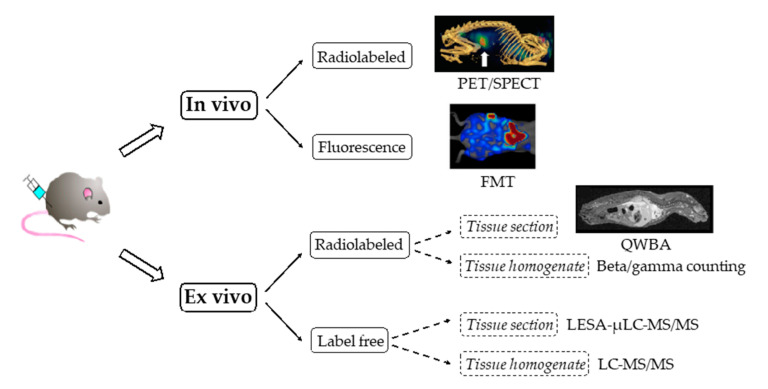
Overview of the different in vivo and ex vivo methods used to study the biodistribution of ADCs in preclinical model. PET: positron emission tomography. SPECT: single-photon emission computed tomography. FMT: fluorescence molecular tomography. QWBA: quantitative whole-body autoradiography. LC–MS/MS: liquid chromatography tandem mass spectrometry. LESA–µLC–MS/MS: liquid extraction surface analysis coupled to liquid chromatography tandem mass spectrometry. PET/SPECT, FMT, and QWBA images were extracted from doi:10.18632/oncotarget.27263; doi:10.1158/1535-7163.MCT-15-1012 and doi:10.1124/dmd.115.069021, respectively.

A label-free method has also been described for the quantification of the Cys-Mc-MMAF metabolite in homogenates of tumor, liver, lung, kidney, and heart (Figure 3). This has been achieved by a LC–MS/MS analysis using the amino acid linker-drug Cys-Mc-MMAD as an internal standard [85]. The direct quantification of the released payload Lys-MCC-DM1 on tumor and liver sections has also been achieved by liquid extraction surface analysis micro-liquid chromatography–tandem mass spectrometry (LESA-µLC–MS/MS, Figure 3) [86].

## 3. Analytical Methods Requiring a Prelabeling of ADCs

### 3.1. Noninvasive Molecular Imaging Approaches

Imaging of radiolabeled ADCs by positron emission tomography (PET), or immuno-PET, has been employed for the noninvasive quantification of ADCs uptake in normal and tumor tissues (Figure 3) [87]. For immuno-PET agents, different radioactive isotopes can be used, including ^64^Cu, ^86^Y, ^89^Zr, and ^124^I. This approach has been notably exploited to challenge the stability of an original bifunctional platinum (II) linker incorporated within the core of ^89^Zr-labeled Trastuzumab, and conjugated to two different payloads (Desferal or auristatin) in a preclinical model [88] or to assess the targeting capacity of novel mAb806-antibody–drug conjugates in malignant mesothelioma [89]. Noninvasive in vivo imaging technologies such as single-photon emission computed tomography (SPECT) have been also used to determine the whole-body biodistribution of ADCs using a ^89^Zr or a ^111^In radiolabeling on the antibody component [90,91,92,93]. An alternative method based on fluorescence molecular tomography (FMT) has been also reported to assess the whole-body distribution of an antibody labeled with a near-infrared fluorophore [94] (Figure 3).

### 3.2. Ex Vivo “Cut and Count” Techniques

The evaluation of ADCs pharmacokinetics and biodistribution in preclinical models can also be performed by either labeling the antibodies component with ^125^I [48,95,96,97], ^123^I [98], or ^111^In [91,97,99,100] or the drug with ^3^H [48,86,101]. In this case, the pharmacokinetics can be obtained by collecting blood samples, within which the content in radioactivity is assessed by liquid scintillation counting (Figure 3). The biodistribution is examined after animal sacrifice at different time points, tissues harvesting and ex vivo “cut and count” techniques enabling to quantify the radioactivity levels in different organs using beta [101,102] or gamma counters [91,92,99,103]. Of note, two examples of quantitative whole-body autoradiography (QWBA) have been recently reported to study the tissue distribution in depth of ^125^I labeled antibody [96], and a ^3^H-labeled DM1 payload [86]. Regarding the latter and beyond the possibility to accurately quantify the amount of payload accumulated within the tumor, the use of liquid extraction surface analysis coupled to micro-liquid chromatography–tandem mass spectrometry allowed to identify a unique catabolite distributed in the tumor and liver tissue.

### 3.3. Dual Radiolabeling

Surprisingly, though the dual radiolabeling of both ADCs components should provide the most comprehensive understanding of ADCs in vivo performances, only a very few examples have been reported to date [102,103,104,105]. The biodistribution of the antibody component and the payload-related species in different organs has been thus described in a case of a ^3^H antibody and ^14^C-labeled MMAF [102], and a ^89^Zr antibody and ^131^I-labeled tubulysin analogue [103]. Digital autoradiography systems can acquire data from multiple radioisotopes and accurately discriminate them based on their difference in emission energies. Thus, the use of cryo-imaging quantitative autoradiography (CIQA) has been described to study the distribution to the tumor of a ^111^In antibody and ^3^H-labeled MMAE, where the ^3^H signal has been acquired after a delay of more than 10 ^111^In half-lives (2.8 days) [104]. More recently, a dual labeling strategy has been used to challenge the stability of the Pt(II)-based linker mentioned above [105]. Of note, dual imaging is not achievable by noninvasive PET imaging since the use of two radioelements at the same time remains challenging mainly due to energy interference.

### 3.4. Limitations of Analytical Methods Involving Radiolabeling

Although allowing a high sensitivity of detection, quantitative radio imaging studies provide no information on the identity of ADC-related species and drug metabolites present in the tissues. To this purpose, these imaging methods must be associated to qualitative bioanalytical methods. In this respect, tissues extraction followed by a SEC-HPLC or standard HPLC analyses coupled to a radioactivity detector can partly circumvent these issues [86,101]. Further, a single labeling on antibody component or payload does not allow to monitor the whole set of ADCs-derived analytes. To overcome these limitations, different and complementary technologies (e.g., liquid scintillation counting, liquid chromatography/mass spectrometry, enzyme-linked immunosorbent assay, and size exclusion chromatography) have to be used to access the pharmacokinetics of the total antibody, the released small molecule drug as well as its metabolites.

Whether it refers to single or dual labeling, these methods all require expensive radioisotopes and dedicated facilities that complicate their clinical transfer. In addition, the radioactive tags are often incorporated randomly within the ADCs core, with potential consequences on their in vivo behavior. In this respect, the incorporated tag may cause immunoreactivity or alter the characteristics of ADCs binding and distribution.

## 4. Conclusions

ADME characterization of ADCs is a complex process due to the multicomponent and heterogeneous nature of these conjugates. While conventional large- and small-molecule methods can be used for ADC bioanalysis, it is however important to understand their limitations to develop ADCs-dedicated bioanalytical strategies.

These recent years, the hybrid LB-LC–MS method has become the most versatile assay in ADCs bioanalysis since they can provide information not only on the entire molecule but also on ADC-derived analytes. When combined with a highly specific LC–MS method, LBA has overcome its intrinsic limitations, mainly related to interferences with highly abundant proteins, to enable unambiguous identification of ADC-derived analytes in many different biological samples. In addition, recent emerging technologies such as an enrichment strategy via pH regulation and ionic solvent strengths [106] are completing the array of LC–MS quantification of biotherapeutics in biological matrices and should be soon applicable to ADCs.

The use of radiolabeled ADCs to support pharmacokinetic and biodistribution studies is undoubtedly a way to reach high detection threshold. More specifically, designing a dual radiolabeled ADC, harboring distinct radioactive tags on the protein component and the small molecule drug, could appear as a particularly valuable strategy to monitor the in vivo fate of both entities simultaneously and to accurately and sensitively measure their relative amount during the circulation and distribution phases. Combined with whole-body imaging, such an approach can provide useful information on overall stability of the conjugate as well as its biodistribution within target-expressing and nontarget-expressing tissues, thus assessing the ADCs efficacy and potential toxicity. However, imaging and accurate quantification by radioactive counting must be associated to qualitative analyses such as HPLC or SEC-HPLC to provide information about the identity of the radio emitting species. Furthermore, the dual radiolabeling must neither interfere with the physicochemical properties of both ADCs components, nor add further heterogeneity that could alter ADCs binding properties and in vivo behavior. Accordingly, the conception of dual labeled ADCs must imperatively rely on efficient radiolabeling reactions that do not affect the drug properties, namely traceless reactions, and on site-specific conjugation reactions aiming at controlling the modification made within the antibody core. Once these limitations are overcome, the dual labeling strategy could become a central methodology to rapidly challenge and optimize various ADCs in preclinical models, thus accelerating their transfer to clinical studies.

## Figures and Tables

**Table 1:** Antibody–drug conjugates (ADCs) approved by the Food and Drug Administration (FDA).

ADC	FDA Approval	Indication	Target Antigen	Antibody	Linker	Payload	Conjugation	Average DAR
Mylotarg^®^	2000, withdrawn in 2010 and reapproved in 2017	Relapsed or refractory acute myeloid leukemia	CD33	IgG4 humanized	Hydrazone (cleavable)	Calicheamicine	Lysine	2.5
Adcetris^®^	2011	Cutaneous anaplastic large cell lymphoma	CD30	IgG1 chimeric	Val-Cit (cleavable)	MMAE	Cysteine	4
Kadcyla^®^	2013	HER2-positive metastatic breast cancer	HER2	IgG1 humanized	SMCC * (noncleavable)	DM1	Lysine	3.5
Besponsa^®^	2017	Acute lymphoblastic leukemia	CD22	IgG4 humanized	Hydrazone (cleavable)	Calicheamicine	Lysine	6
Polivy^®^	2019	Diffuse large B-cell lymphoma	CD79b	IgG1 humanized	Val-Cit (cleavable)	MMAE	Cysteine	4
Enhertu^®^	2019	HER2-positive metastatic breast cancer	HER2	IgG1 humanized	Gly-Gly-Phe-Gly (cleavable)	DXd	Cysteine	7.5
Padcev^®^	2019	Locally advanced or metastatic urothelial cancer	Nectin-4	IgG1 fully human	Val-Cit (cleavable)	MMAE	Cysteine	3.8
Trodelvy^®^	2020	Triple negative breast cancer	Trop-2	IgG1 humanized	Carbonate (cleavable)	SN-38	Cysteine	7.6
Blenrep^®^	2020	Relapsed or refractory multiple myeloma	BCMA	IgG1 humanized	MC * (noncleavable)	MMAF	Cysteine	4

* SMCC: Succinimidyl 4-(*N*-maleimidomethyl)cyclohexane-1-carboxylate; MC: Maleimido-caproyl.

## Abbreviations

ADA	Antidrug antibody
ADC	Antibody–drug conjugate
ADME	Absorption, distribution, metabolism and excretion
CIQA	Cryo-imaging quantitative autoradiography
DAR	Drug-to-antibody ratio
DM1	Mertansine
DM4	Ravtansine
DOTA	Tetraxetan
DTPA	Pentetic acid
EIC	Extracted-ion chromatogram
ELISA	Enzyme-linked immunosorbent assay
EPR	Enhanced permeability and retention
Fab	Fragment antigen binding
Fc	Fragment crystallizable
FcRn	Neonatal Fc receptor
FDA	Food and Drug Administration
FMT	Fluorescence molecular tomography
HER-2	Human epidermal growth factor receptor 2
HR/AM	High resolution accurate-mass
HRMS	High resolution mass spectrometry
HRP	Horseradish peroxidase
IgG	Immunoglobulin G
IS	Internal standard
LBA	Ligand binding assay
LB-LC–MS	Ligand-binding immuno-affinity capture followed by LC–MS analysis
LC–MS	Liquid chromatography tandem mass spectrometry
LESA–µLC–MS	Liquid extraction surface analysis coupled to µLC–MS
LLOQ	Lower limit of quantification
MMAE	Monomethyl auristatin E
MMAF	Monomethyl auristatin F
nSMOL	Nano-surface and molecular-orientation limited proteolysis
PET	Positron emission tomography
QWBA	Quantitative whole-body autoradiography
SEC	Size exclusion chromatography
SHA	Semihomogeneous assay
SPE	Solid phase extraction
SPECT	Single-photon emission computed tomography
T-DM1	Trastuzumab emtansine/Kadcyla^®^
